# Assessing organisational capacity for evidence-informed health policy and planning: an adaptation of the ORACLe tool for Australian primary health care organizations

**DOI:** 10.1186/s12961-021-00682-5

**Published:** 2021-02-18

**Authors:** Alice Windle, Sara Javanparast, Toby Freeman, Fran Baum

**Affiliations:** 1 College of Medicine and Public Health, Southgate Institute for Health Society and Equity, Flinders University, Adelaide, SA Australia; 2 Discipline of General Practice, College of Medicine and Public Health, Flinders University, Adelaide, SA Australia

**Keywords:** Evidence-informed health planning, Policy-making, Organizational capacity, Primary health care, Oracle tool

## Abstract

**Background:**

Many nations have established primary health care (PHC) organizations that conduct PHC planning for defined geographical areas. The Australian Government established Primary Health Networks (PHNs) in 2015 to develop and commission PHC strategies to address local needs. There has been little written about the capacity of such organizations for evidence-informed planning, and no tools have been developed to assess this capacity, despite their potential to contribute to a comprehensive effective and efficient PHC sector.

**Methods:**

We adapted the ORACLe tool, originally designed to examine evidence-informed policy-making capacity, to examine organizational capacity for evidence-informed planning in meso-level PHC organizations, using PHNs as an example. Semi-structured interviews were conducted with 14 participants from five PHNs, using the ORACLe tool, and scores assigned to responses, in seven domains of capacity.

**Results:**

There was considerable variation between PHNs and capacity domains. Generally, higher capacity was demonstrated in regard to mechanisms which could inform planning through research, and support relationships with researchers. PHNs showed lower capacity for evaluating initiatives, tools and support for staff, and staff training.

**Discussion and conclusions:**

We critique the importance of weightings and scope of some capacity domains in the ORACLe tool. Despite this, with some minor modifications, we conclude the ORACLe tool can identify capacity strengths and limitations in meso-level PHC organizations. Well-targeted capacity development enables PHC organizations’ strategies to be better informed by evidence, for optimal impact on PHC and population health outcomes.

## Background

The importance of using evidence to inform health policy and planning decisions is well recognized. These decisions are also influenced by practical, political and ideological factors. Much research has sought to identify key barriers to the ‘push’ and ‘pull’ of evidence for use in health policy-making [1], which can be broadly categorized as follows: decision-makers lack access to appropriate evidence; or the need to balance a complex range of political, ideological or other influences, with evidence [2–4]. Following earlier calls to increase capacity to use evidence in decision-making [5], further similar calls have been made in recent years [6–10] and has led to the development of various tools to assess health policy agencies’ organizational capacity for using research. The most recent of these, building on lessons from earlier tools, is the ORACLe interview tool which examines seven domains of organizational capacity, and was developed by the Sax Institute in Australia, within a suite of tools and services to help policy agencies improve evidence-informed policy-making [8, 11, 12]. One such tool is the SAGE tool, which is based on qualitative assessment of research use in policy development, and has been used alongside the ORACLe tool [12]. The ORACLe tool has been assessed as methodologically sound [13], and has been applied to policy agencies in Australia [7].

Extensive literature on the use of evidence in decision-making spans a broad range of disciplines, and health policy-making and clinical practice are areas that feature prominently. In between these realms of broad population-based approaches implemented by governments, and individual services delivered by health care professionals is the meso level of health planning, which involves devolved, relatively autonomous regional decision-making for a geographically defined population. While not strictly ‘policy-making’, it similarly involves making decisions and allocating public money to interventions designed to improve population health, amidst a complex range of competing influences. Devolved decision-making, as with that of ‘higher’ levels of government, is likely to be most useful and least harmful when informed by the systematic and transparent use of a range of different types of evidence [14, 15], yet there is a paucity of literature exploring the use of evidence in this context.

Meso-level, regional primary health care organizations (PHCOs) feature in the health systems of numerous high-income countries, including the United Kingdom [16], New Zealand (Primary Health Organizations, [17]) and Scotland (local authorities and Integrated Joint Boards, [18]). In Australia, there are 31 Primary Health Networks (PHNs), funded by the Australian Government, with responsibility for allocating hundreds of millions of (public) dollars to primary health care initiatives to improve health outcomes and access. However their capacity to achieve these goals has been queried [19] and so examination of their capacity for evidence-informed planning is warranted.

This paper provides a reflective critique of the use of a slightly adapted version of the ORACLe tool to assess the organizational capacity of meso-level PHCOs for evidence-informed health planning critically, and presents the findings of applying the tool to Australian PHNs.

## Methods

### Research context

This research extends on a larger National Health and Medical Research Council-funded mixed-methods research programme that ran from 2014 to 2018, and examined various aspects of Australian PHCOs (PHNs and their predecessors, Medicare Locals): governance, health equity, comprehensive PHC approaches, population health planning, evidence use in planning and more. The research has employed a range of methods; however, the principal approach drawn on in this paper has been case studies of five PHNs. A purposive sample of PHNs were recruited, ensuring a range of metropolitan and rural/remote PHNs from different states/territories. Four of the PHNs had participated in earlier stages of the research, and another was recruited on the basis of similarities with a discontinuing PHN. Two of the five PHNs were in metropolitan areas, one was in a rural area, and two covered both metropolitan and rural areas. To ensure anonymity, participating PHNs are referred to as Metro North, Metro South, Rural North, Rural South, and Remote.

This paper focusses on data drawn from 14 semi-structured interviews with employees from participating PHNs, conducted in 2018 specifically for this component of the research. The interviews also included a range of questions on other factors relevant to evidence-informed planning. Analysis of internal policy documents supplemented the interview analysis. Qualitative analysis of all interviews was conducted alongside ORACLe tool analysis.

### ORACLe tool: adaptation and interview protocols

The ORACLe tool is an interview schedule of 23 questions, designed to examine organizational capacity for evidence-informed policy-making in health policy agencies. (For a detailed description of the development and validation of the ORACLe tool, see Makkar et al. [8].)

The tool examines capacity across seven domains, each weighted for their relative importance:

1. Documented processes to develop policy and plans that encourage or mandate the use of research (11.88%)
2. Tools and programmes to assist leaders of the organization to actively support the use of research in policy and programme development (19.48%)
3. Availability of programmes to provide staff with training in using evidence from research in policy and in maintaining these skills (20.53%)
4. Availability of support and tools to help staff access and apply research findings (17.57%)
5. Presence of systems/methods to generate new research evidence to inform the organization’s work (8.74%)
6. Clear methods to allow adequate, evidence-informed evaluations of the organizations’ policies and programmes (10.96%)
7. Mechanisms that help strengthen staff relationships with researchers (10.84%) [8]

The ORACLe tool questions were incorporated into a semi-structured interview schedule that addressed a range of issues related to evidence-informed, equity-focussed health planning, including organizational capacity.

In order to maintain the established validity of the tool, little change was made to the content and intent of the questions, other than to adapt some wording to reflect PHN planning, rather than government policy-making. For example, questions that originally specified ‘in the last 6 months’ were changed to ‘12 months’ to reflect the annual commissioning cycle of PHNs. In preparing the ORACLe-based interview schedule, the scoring guide was consulted to help ensure that further ‘probe’ questions were included so that sufficient detail could be obtained for scoring. Given that interviews were semi-structured, there was some variation as to the use of further probing questions, as necessary, and sometimes in the ordering of questions, in response to the ‘flow’ of the more conversational style of interviews. The interview schedule was piloted with two interviewees from a non-participating PHN.

Participating PHNs were invited to nominate interview participants, representing three different levels of involvement in planning and programme development—CEO (or deputy), and a senior manager and staff member involved in planning. One PHN nominated only two participants because they only had a small team to draw from. None of the invited interviewees declined. All interviewees gave informed consent to participate in the research, and none dropped out. Of the 14 interviews, 13 were conducted face-to-face at the respective PHN, and one was conducted via telephone. Interview duration ranged from approximately 60 to 80 min. There were no non-participants present in interviews. Interviews were conducted between May and September 2018, and there were no repeat interviews. Interviews were conducted by AW, a female PhD candidate who has experience in qualitative interviewing, and has worked for PHCOs in planning. Two of the interviewees had prior professional peer interactions with the interviewer, and the rest had only a preliminary introduction to the research and interviewer prior to participation. At the beginning of each interview, the interviewer introduced herself and provided a summary of her experience in PHCOs and the research aims. All interviews were digitally recorded and professionally transcribed. Field notes were also made during and after each interview. All interviewees were offered the opportunity to review their transcript prior to analysis.

Ethics approval was granted by the Flinders University Social and Behavioural Research Ethics Committee (Approval #6376), and all participants gave informed consent to participate.

### ORACLe tool analysis and scoring

Transcripts were coded using NVivo qualitative analysis software (QSR, Doncaster, Victoria), using a coding framework of key research themes drawn from a conceptual framework of evidence-informed health policy-making [5], and the specific ORACLe tool questions.

The original application of the ORACLe tool involved only one CEO interview per organization, which made for simple allocation of a score based on the responses from one person. As recognized in the open peer review of the ORACLe paper [20], and by others [21], perceptions can differ between individuals within the same organization, so our research drew on 2–3 interviewees from each organization.

Once coded to relevant nodes, ORACLe data were extracted from NVivo into a MS Word table, with responses organized by question for each PHN. The approach to scoring each question involved first consulting the scoring guide for the respective question [8], and then doing a ‘familiarisation’ read through all relevant responses. A second closer reading of each response was then conducted, and a preliminary score allocated to each individual’s response. Responses were then re-read, to check for consistency within the PHN. A corresponding ‘consensus score’ for the question, for the PHN was assigned. Where scores were consistent, this became the consensus score, and where there were inconsistent responses between interviewees, judgment on the most valid response was made, on the basis of being more detailed, or a more relevant perspective. For example, the planning manager was deemed to be best placed to know whether their position description covered expertise in use of research in planning (Question 4). Throughout this process, there was frequent comparison and checking between PHNs, to ensure a consistent approach. The rationale for assigning the consensus score was noted in the table. Qualitative thematic analysis of data was also conducted alongside ORACLe scoring, the detailed findings of which will be reported elsewhere.

While the original ORACLe tool involved scores of only whole numbers (1, 2 or 3), this application of the tool allowed for increments of 1.5 and 2.5 where the response was greater than the lower score, but did not qualify for the higher score, according to the scoring guide. Intermediate scores have been used in other applications of the tool [7]. Intermediate scores were assigned for only 14 of the 130 responses. The ORACLe tool paper recommended that scoring be conducted by an independent person, who had not conducted interviews [8]. In this research, AW conducted, coded and scored the interviews. A scoring validity check on responses from one PHN was conducted by a member of the research team (TF), which indicated a satisfactory degree of consistency. Differences were discussed until agreement was reached.

Once consensus scores had been assigned for all questions and PHNs, scores were entered into a MS Excel spreadsheet. Total and average question consensus scores within capacity domains were calculated. Total weighted scores for each PHN were also calculated using the conditional logit model outlined in Additional File 1 of the ORACLe paper [8].

### Internal document analysis

Thirty internal policy or guidance documents were sourced. Twenty-six were provided by PHNs and four were downloaded from their websites. These documents were examined for documented planning processes/procedures. This evidence was then triangulated with interview responses regarding documented processes, from Domain 1 of the ORACLe tool. While this aspect was not specified in the original ORACLe procedure, we collected these data to add rigour.

### ORACLe tool critique

Before using the ORACLe tool in this research, a theoretical critique was conducted to examine its alignment with key theory in the international literature on evidence-informed health policy-making and to consider its appropriateness for application to PHCOs. This critique is examined in the “Discussion” section of this paper. While some concerns were identified, it was decided that the tool was acceptable to use.

A practical critique of the ORACLe was based on the reflections of the first author’s experience in piloting the tool, using it in interviews and subsequently coding data and assigning scores. This drew on the field notes taken during and after interviews, and a methodology journal kept during the process of data coding and analysis.

## Results

### PHN scores and capacity

**Fig. 1:**
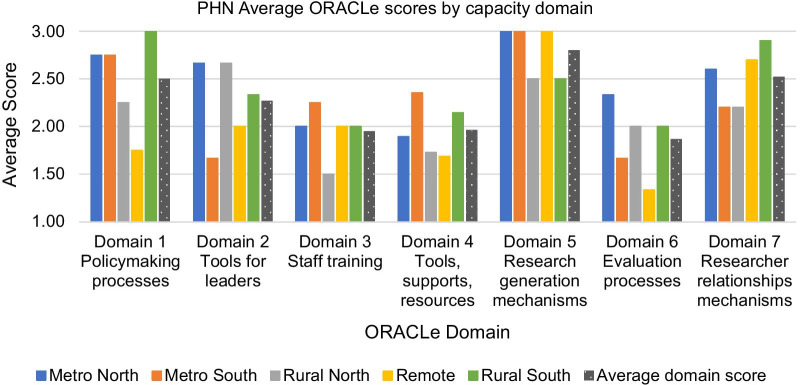
PHN average scores by capacity domain

Application of the ORACLe tool identified variation between PHNs and between capacity domains. On the basis of unweighted, average domain scores, no one PHN consistently scored higher or lower than others (Fig. 1).

PHNs generally demonstrated moderate to high capacity, based on average domain scores ranging from 1.9 to 2.8 (out of 3).

Strongest capacity was demonstrated in generating new research, through frequent, recent internal research such as focus groups, surveys, and data analysis, or through externally commissioned research projects (Domain 5).

Several PHNs demonstrated moderately strong capacity through the reported existence of documented processes that provide detailed guidance on planning/programme development, and explicitly encourage or require evidence use (Domain 1). PHNs also demonstrated moderate to strong capacity in terms of mechanisms for relationships with researchers, by virtue of having both formal and informal relationships and participation at conferences, and to lesser extents, researcher participation in advisory groups, and joint appointments with research organizations (Domain 7).

Moderate capacity was demonstrated in relation to leadership supports for the use of evidence, in that for most PHNs, leaders’ position descriptions explicitly covered expertise in use of research, and generally there was relevant professional development available for leaders. There was variation in the extent to which leaders reported mentioning research/evidence in their internal communications (Domain 2).

There were three capacity domains for which average scores sat below the midpoint of 2, indicating relatively lower capacity. Training for staff was generally available and considered in performance management, although mainly not specifically regarding evidence use, and was ad hoc (Domain 3). PHNs’ supports for accessing and applying research evidence varied considerably. Staff with such expertise were relatively common, but there was variation in the extent to which relevant research was internally disseminated, or there were subscriptions to research journals and databases, or documented methods to commission reviews. There was generally low capacity in terms of knowledge management systems, libraries, reference management software, and particularly resources to guide the use of research evidence (Domain 4). While PHNs generally encouraged evaluation to be built into programme development, either implicitly or explicitly, capacity was lower in terms of documented, evidence-informed processes for conducting evaluation (Domain 6).

PHNs’ scores tended to be lower in the domains with high importance weightings (2, 3 and 4) and higher in the lower weighted domains (1, 5 and 7).

**Fig. 2:**
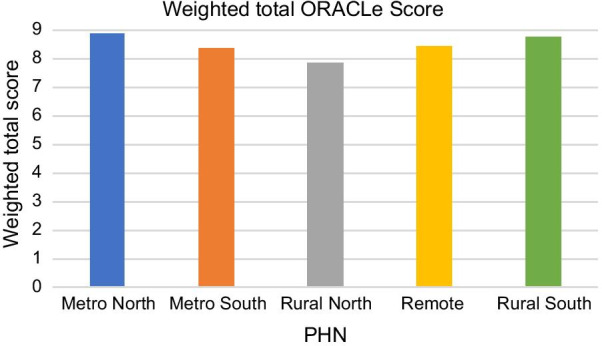
Weighted total ORACLe scores

When recommended weightings were applied and domain scores totalled [8], there was less variation between PHNs, and all PHNs scored highly, indicating strong capacity (range 7.8–8.9 /9) (Fig. 2).

### Assessment of ORACLe tool

Key strengths and limitations of the usability and value of the ORACLe tool, in meso-level PHC planning are outlined below.

### Strengths

The decision to allocate scores on the basis of several interviews including the CEO was warranted, as there were indications in several instances where the CEO was not best placed to respond, for example:

“Probably [manager] would be better able to inform you as to what happens with that stuff [various evidence sources/materials] when I send it through to them. I have to say I don’t know what happens to an awful lot of the stuff that I push down” (Senior Executive, Rural South PHN).

An assessment of all interviews and questions suggests there were six responses for which there was insufficient data to allocate a score for one of the respondents. In these cases, it was valuable to have other responses to refer to. There was complete agreement between responses from all participants within a PHN on around half of the questions. There was also a degree of disagreement, which partly reflected the varying detail of responses, as well as the different perspectives and knowledge of interviewees.

Examination of PHN internal documents supported the favourable interview data regarding documented processes that encourage or require evidence use (Domain 1). The various documents examined tended to include broad guidelines for planning, such as overviews of the general commissioning cycle, and very few included specific procedures for programme planning, as the ORACLe tool sought. The documents did not include any prompts or mechanisms by which to critique the appropriateness or transferability of evidence for the issue under consideration. The most relevant documents were comprehensive commissioning toolkits or manuals (three PHNs), and templates for activity or programme plans (three PHNs). There was much to indicate that use of evidence was encouraged, but only a few examples of an evidence base or rationale being documented.

When there were several criteria involved in scoring a response, it was more difficult to allocate a score, and the ability to allocate an intermediate score (1.5 or 2.5) was helpful. For example, four PHNs were allocated a score of 2.5 on the question of whether the PHN has documented processes for how plans/programmes should be developed (Question 1, Domain 1). To achieve the full score of 3, there had to be detailed, organization-specific written guidance. In each of these PHNs interviewees were not confident in the degree of detail, so while there were documents, developed by that PHN, the full score was not warranted because of documentation lacking detail.

Our inclusion of additional ‘probe’ questions into the interview schedule proved to be essential to obtain sufficient detail for allocating scores to the responses.

### Challenges

The existence of documented processes that encourage the use of evidence is a positive and important aspect of organizational capacity. However, the value of such documents relies on how well the guidance is applied or the processes adhered to. Despite high scores in this domain (1), it was reported by interviewees (from a high scoring PHN) that documented processes were not consistently followed because they were too detailed, and their use was not actively encouraged. Some inconsistency between interview responses within this PHN suggests that while documented processes exist (and a high score is warranted), they are not consistently used.

Our application of the ORACLe tool raised some general issues concerning its structure, flow and wording. For example, there was some overlap of questions and concepts between different domains, which made some aspects of the interview seem repetitious and disjointed. For example, there were two nonconsecutive questions about training for leaders (Domain 2) and training for staff (Domain 3), yet interviewees tended to talk about them all together. However, using NVivo, responses could be coded (and scored) to the relevant domain regardless of which question was being answered.

Some questions were commonly misinterpreted by interviewees, suggesting they may need to be worded differently. For example, Question 5 (Domain 2) asked about leaders *referring* to research/evidence in their internal communications, whereas four interviewees responded about *using* evidence in planning/programme development. Several questions may have benefitted from a preamble statement or preliminary question to clarify the focus.

Some questions, as worded, were not appropriate to the PHCO context. For example, Question 11.3 asked about having a library, which while reasonable in a large government department, is unlikely in a small organization. Subtly changing the question to ask about *access to* a library may have been appropriate. Some interviewees’ responses implied that they perceived certain questions as unrealistic: *“Um, goodness me…”* (Senior Executive, Rural South PHN).

In some domains, the scores for certain questions were dependent on responses to other questions. For example, if 1 was scored for Question 17 (documented processes for evaluation), 1 was the only possible score for Question 18 (evaluation processes based on research) which potentially skewed results in this domain, which had the lowest average domain score, and considerable range (1.33–2.33).

In Questions 20 and 21 (formal and informal relationships respectively), the score was based on the number of relationships, which seems a somewhat crude way to measure engagement with researchers, and does not examine mechanisms to facilitate relationships.

Deeper probing for detail may have been beneficial in some questions. Many of the questions, as written in the original interview schedule, did not include sufficient probes to obtain the level of detail required by the scoring guide. Better interviewer technique to pursue the appropriate level of detail may also have helped; however, this can be challenging in a semi-structured interview where the order of questions can vary. Maintaining rapport with the interviewee and not being too assertive in asking critical questions is also an important consideration.

A criticism of the original tool was the use of the term ‘research’, and in practice, wording was sometimes adapted to reflect the broader concept of ‘evidence’. Some interview participants’ responses also explicitly distinguished between ‘research’ and ‘evidence’:

“Evidence yes. Research hmm?!” (Manager, Metro South),

“Aah, I don’t know if it’s the use of research, it’s about being evidence-based” (Manager, Remote)

Although not recommended in the original ORACLe tool, qualitative analysis of ORACLe and additional interview data added to the ORACLe results provided a richer understanding of planning processes and influences on capacity. This analysis also identified ongoing strategies to improve aspects of capacity that did not yet strictly qualify the organization for a higher score. For example, one PHN was in the process of developing an evaluation framework, but did not yet have a documented guide for evaluating programmes. Qualitative analysis also identified a broad range of relationships between PHNs and other organizations, and various direct and indirect influences these have in evidence-informed planning. For example, strong relationships with state/territory government health departments enabled access to hospital and other data, which were an important evidence source for PHNs.

## Discussion and conclusions

Our application of the ORACLe tool demonstrated it could be used to identify strengths and weaknesses in PHCOs’ organizational capacity for evidence-informed planning. This section will discuss the utility of the tool for this purpose, explain some modifications we made, and explore criticisms we identified, reflecting on some key theoretical considerations regarding capacity elements and hierarchy.

### Application to meso-level organization

Our research indicates that the ORACLe tool, while developed for state or national health policy-making, can be applied to examine organizational capacity for evidence-informed decision-making in meso-level health planning organizations. Within organizations it can help to identify certain aspects of capacity that may benefit from further development. It also offers value in potentially identifying common capacity limitations across like organizations, which may be addressed by broader capacity development strategies.

Our findings suggest that PHNs’ capacity, based on adjusted weighted domain scores, is comparable, if not slightly stronger than that of government policy agencies [7]. PHNs generally demonstrated stronger capacity in Domain 2 (tools for leaders), and Domain 5 (research-generating mechanisms), and somewhat lower capacity in Domain 4 (tools, supports, resources). Any comparison between different types of policy agencies should be undertaken with caution, however, as they are subject to different contextual influences on their capacity, particularly autonomy and funding volumes. Our application of the ORACLe tool also identified some wording and content aspects of questions that meant they were potentially less relevant to regional health planning organizations than the ‘higher’ policy level for which the tool was designed. It is important to interpret capacity findings relative to the context in question.

### Modifications of ORACLe tool employed in this research

While this research indicates that ORACLe is valuable in examining capacity strengths and weaknesses, it does not allow for a qualitative understanding of why capacity might be limited, or progress in developing capacity. We found that qualitative analysis can complement ORACLe findings in these respects, as well as allowing a broader exploration of related themes.

We also undertook some document analysis to complement the ORACLe tool which was helpful in corroborating Domain 1 findings. Further document analysis (e.g. position descriptions) may have helped to validate responses to other relevant questions. While the ORACLe tool examines the existence and content of documents, it does not allow for examination of the extent to which documents are used or reflect practice. It also does not go to the detail of examining if/how evidence is appraised and applied. A more comprehensive ‘audit’-type process may yield more valid findings about organizational capacity and process.

This research was strengthened by administering the ORACLe tool with several participants from each organization, instead of only the CEO. It helped to overcome some paucity of data, either through insufficient probing or interviewee knowledge gaps. If detail was lacking from one interview, it could be obtained from another. It did complicate the scoring in some instances, where responses differed and judgement was required; however, we consider this made for a more accurate result. We also found that inconsistent responses within an organization can indicate issues with internal policy implementation, in that documented guidance, while it existed, was not necessarily followed.

Our inclusion of additional ‘probe’ questions was essential to obtain sufficient detail to allocate scores to responses. That these additional questions are not necessarily consistent in other applications of the tool is a further reason for exercising caution when comparing PHN capacity findings from this research, with capacity studies of other organizations.

### Interview question terminology and adjustments

Our experience highlighted one concern with the ORACLe tool in the use of the term ‘research’, which reflects, and potentially constrains responses to a narrow conception of evidence. While much of the evidence-informed policy literature focusses on research evidence generated by academics and published in peer-reviewed journals, it is frequently recognized that decision-makers’ conceptions of evidence will differ from those of academic researchers [3]. A wide variety of academic and non-academic information sources from a range of disciplines inform policy-making and planning [22]. The types of information used in health planning fall into six broad categories: demographic, epidemiologic, health services activity, health economic, stakeholders’ qualitative data and intervention evidence (‘what works’) [15]. Conceptions and use of evidence differ depending on the context or policy ‘level’ in which decisions are made. Locally appropriate evidence such as stakeholder consultation and local service utilisation data is more likely to be used in meso-level regional planning. The focus of the ORACLe tool on ‘research’ and the inconsistent substitution with the term ‘evidence’ is potentially a limitation of the current version of the tool. One possible strategy would be to provide a definition of a broad conception of evidence at the beginning of the tool, and then use consistent terminology throughout.

We suggest that the order and interpretation of questions may benefit from some rearranging, or a preamble, to clarify the focus of some questions. Some interdependence between questions for allocating scores was identified and may need to be addressed, such as the questions regarding evaluation processes, as outlined above. Some questions may also benefit from minor wording changes, to make them more applicable to small–medium, meso-level organizations with annual planning cycles, rather than large government agencies.

### Narrow examination of network capacity

Domain 7 of the ORACLe tool examines capacity regarding staff relationships with researchers. Our broader qualitative analysis identified a range of important relationships in addition to those with researchers, indicating that this domain of the ORACLe tool is potentially too narrow. While this domain of the ORACLe tool is based on a wealth of sound evidence that relationships with researchers facilitate evidence use [8], an expanded examination of ‘communication and networks’ with a range of stakeholders may provide a more comprehensive assessment of this component of organizational capacity than the narrower focus on relationships with researchers. The focus on relationships with researchers strongly reflects the ‘two communities’ theory [23] that use of evidence in policy-making is hindered by researchers and policy-makers being two distinct communities, with different ‘norms’ and drivers for their actions and priorities. The ‘two communities’ theory has been increasingly criticized as overly simplistic, and the complex relationships or networks between researchers, policy-makers and other actors are influential in the policy environment, more richly explained by the ‘advocacy coalition framework’ [24]. A further criticism in this regard is that the Domain 7 questions tend to focus on the existence of relationships (in a given time period), and less so on the existence of mechanisms or structures to establish or maintain those relationships. There is also no examination of the quality of relationships, or the degree of influence of external stakeholders on evidence-informed planning. Just as research is one of many sources of evidence for policy/planning, our research indicated other relationships are also important enablers of evidence-informed decision-making particularly in regional planning, and a broader examination of this capacity would strengthen the ORACLe tool.

### What about governance capacity?

Governance is recognized as an element of organizational capacity for evidence-informed policy/planning [5] yet was given relatively little attention in the ORACLe tool. ‘Good governance’ identifies and manages what political science theory explains as the range of competing ‘political’ interests, values and other influences on policy. Hawkins and Parkhurst [2] recommend a ‘good governance’ framework that examines the *process* of evidence-informed policy-making as opposed to the *outcomes* of policy-making, against the principles of appropriateness, transparency, accountability and contestability.

While Domain 1 partly addresses governance by examining the existence and detail of ‘documented processes’, the assessment of governance capacity for evidence-informed decision-making in the ORACLe could be strengthened by more detailed examination of systems to embed key governance principles. In particular, appraisal of the appropriateness or transferability of evidence is important in meso-level regional health planning, to ensure that the strategies for which evidence is considered are appropriate for the region or community in question. A hypothetical example might be where a strategy to enhance the cultural safety of services for Aboriginal and Torres Strait Islander people has been favourably evaluated in X region—can it then be confidently assumed that the same strategy would be culturally safe in Y region (acknowledging the distinct cultural beliefs and practices of different Aboriginal communities across Australia)?

### Importance weightings of capacity domains

A further issue with the ORACLe tool relates to the importance weightings of capacity domains. Domain weightings (as detailed in the “Methods” section of this paper) were developed on the basis of interviews with senior national and state health policy-makers [8], rather than meso-level planning actors, who potentially rate capacity priorities differently. For example, Domain 5 (presence of systems/methods to generate new research evidence to inform the organization’s work) had the lowest importance weighting, yet this research found that PHNs invest considerable effort and resources into locally appropriate research and stakeholder consultation, which suggests they believe this is of high importance.

We found a generally inverse relationship between domain weightings and capacity scores in this research. An example of this in our research was in relation to the low weighted Domains 5 and 6, which examined organizations’ ability to generate evidence through research and evaluation respectively. While these domains may not appear to be directly linked to capacity to use evidence, it has been argued that analysis performed by bureaucrats within policy organizations is more likely to influence policy than academic research [25], recognising that generation and use of evidence does not occur in distinct organizations [5]. These domains also possibly indirectly indicate the culture and skill base within an organization. If there are staff and processes for generating research and evaluation evidence, there is likely to be ‘research literacy’ within the organization and a culture that values and supports evidence utilisation. In meso-level health planning organizations, where peer-reviewed research literature is less likely to be directly relevant, the capacity of organizations to generate evidence that is contextually relevant and appropriate is likely to be more important. Decision-makers in meso-level organizations may have given these domains a higher importance weighting, and PHNs would likely have achieved stronger results from the ORACLe tool.

Because the weighting may be less valid, the combined weighted capacity scores may also be less valid at the meso-level health planning context than they would be for ‘higher’-level policy agencies.

As well as concerns with the change in context, we also had general theoretical concerns with the weightings specified by the tool. For example, weightings tended to favour support for individual capacity within an organization, with lower weighting assigned to those domains which incorporate systematic or mechanistic approaches to organizational (social) structures and systems. This echoes a key challenge in public health: the persistent adoption of individualistic, behavioural health promotion strategies, rather than more effective population-based, systemic approaches to improve public health [26]. Green and Bennett [5] advocate a ‘systems approach’ to capacity development that attends to organizational processes and the enabling environment, not only skills. In contrast, domain weightings in the ORACLe tool are considerably higher for the domains that reflect a focus on individuals’ skills and tools (Domains 2, 3 and 4), and lower on the domains that address organizational systems and mechanisms (1, 5, 6 and 7). For example, Domain 2 addresses the important attribute of leadership, but examines the individual capacity of leaders being built through tools and programmes. If the examination of ‘tools and programmes to assist leaders’ meant decision matrices or criteria for decision-making this would positively reflect systems to lead evidence-informed planning. However, the questions focus on mechanisms to encourage and develop the “confidence and expertise” of individual leaders in research use. While it is important that leaders have relevant technical knowledge, the capacity of leaders to drive evidence-informed decision-making processes, potentially through systems or governance, may be a better indicator of organizational leadership capacity for evidence-informed planning. Leadership is a broad concept, and can also encompass ‘invisible’ elements within an organization, such as a clear vision and organizational ‘attitude’ [5]. Such intangibles may be loosely inferred from the examination of leaders’ internal research dissemination, but this aspect of leadership capacity is otherwise somewhat neglected. We would argue that rather than disseminating research/evidence within an organization, the responsibility of a leader would be to employ leadership strategies that encourage or require use of research in planning.

It is recognized that development of systems and structures in an organization is more difficult and time consuming than developing individual skills or tools. Potter and Brough [27] argue that capacity building that addresses systems and structures is more important, yet more complex and abstract, with a sociocultural basis. Capacity building that addresses skills and tools is more tangible, measurable and quick, with a technical basis. As such, examinations of capacity can tend to drift away from holistic analysis of a system, towards a simpler focus on individuals [27].

An alternate weighting system, informed by theory, and/or appropriate to the meso-level planning context would likely produce a more valid assessment of PHNs’ capacity for evidence-informed health planning. As it stands, it would not be valid to use the ORACLe tool to compare organizations from different decision-making contexts. However, comparison of unweighted domain scores can be useful to indicate capacity shortcomings within an organization, and also between similar organizations. We would argue against weighting the different domains as to their overall importance, and instead focus on the applicability of the tool to identify specific areas for capacity development within organizations.

### Limitations of this research

The developers of the ORACLe tool recommend that data coding and scoring not be done by the same person who conducts interviews [8]. One of the limitations of this study was that the interviewing and coding/scoring was conducted by the same researcher, in contrast to the conduct of the ORACLe tool. As this was part of a PhD research project, limited resources and capacity prevented having multiple people involved in these core components of the research. However, we note that the cross-checking by a supervisor (TF) helped to mitigate any bias that this may introduce.

Another limitation of this research was that there was no participant checking of the ORACLe scores and findings; however, we feel this was mitigated by drawing on responses from multiple interviewees in assigning scores.

The ORACLe tool was developed and intended to be used alongside the SAGE tool [12] which examines how evidence is used to inform policy. Use of the SAGE tool alongside the ORACLe tool may have helped overcome some of our criticisms of it, and provided a more comprehensive understanding of PHNs’ evidence informed planning and capacity therefor. We did not use the SAGE tool, as to do so would have greatly increased the scope of the research project, for which we had limited resources and capacity.

The approach taken in this research did not allow for direct comparison between the ‘standard’ ORACLe methodology as described by its creators [8] and the adapted approach we employed—to do so may have compromised the comparability of capacity findings between PHNs. However, we do recognize this limitation in the ability to draw comparisons between the standard and adapted tool within this context.

## Conclusion

Competence in policy and planning decision-making is just as important at the meso level as it is at higher levels of government, and capacity for evidence-informed decision-making is a key aspect of such competence.

Through this research, we have demonstrated that the ORACLe tool can be useful to examine aspects of organizational capacity for evidence-informed planning in meso-level PHC organizations. Our application of ORACLe has identified some opportunities to refine or complement the tool, which are outlined in Box 1. While caution should be exercised in comparing capacity between different types of organizations, this tool can potentially be applied within organizations to identify areas for capacity development, or to identify common capacity limitations across like organizations, to inform broader capacity development strategies. Such use of the tool would enable meso-level PHC organizations’ decisions to be better informed by evidence, and maximize the effectiveness and efficiency of strategies and their impact on PHC and population health outcomes.

Box 1 Recommendations to enhance the ORACLe tool:

- Collect data from several individuals within the same organization, to improve the validity of findings.
- Include additional questions to probe for detail.
- Undertake qualitative analysis of broader interview data to provide deeper understanding of capacity limitations.
- Examine relationships and networks more broadly than those with researchers.
- Include additional interview questions and document analysis to examine capacity for leadership and governance of the decision-making process.
- Analyse internal organizational documents to triangulate interview findings.
- Look at a ‘higher’ level of capacity—at the ‘systems and structures’ rather than tools and skills, and if weightings are to be used, they should reflect this.

To improve the appropriateness of the tool for meso-level planning:

- Adopt and make explicit a broad definition of ‘evidence’ that includes material from non-academic sources, rather than ‘research’, and use this terminology consistently.
- Include some examination of mechanisms to assess the appropriateness of evidence to the context in question.
- Adjust wording slightly to make some questions more relevant (for example, is there *access to* a library?).

## Data Availability

Deidentified data can be obtained from the corresponding author on request.

## Abbreviations

PHCO: Primary health care organization
PHC: Primary health care
PHN: Primary Health Network
